# Supplementary material for: vcfpp: a C++ API for rapid processing of the variant call format
Source: Bioinformatics. 2024 Jan 25;40(2):btae049. doi: 10.1093/bioinformatics/btae049 (PMC10868310; doi:10.1093/bioinformatics/btae049)
Supplement: btae049_Supplementary_Data [file btae049_supplementary_data.pdf]

```r
library(vcfppR)
library(parallel)
par(mfrow = c(3,1), mar = c(5,5,5,2), cex.lab = 2, cex.axis
    = 2, cex.main = 2)
vcffile <-"https://ftp.1000genomes.ebi.ac.uk/vol1/ftp/data_
    collections/1000G_2504_high_coverage/working/20201028_
    3202_raw_GT_with_annot/20201028_CCDG_14151_B01_GRM_WGS_
    2020-08-05_chr21.recalibrated_variants.vcf.gz"
vcf <- vcftable(vcffile, region="chr21:1-10000000", samples=
    "NA12878,HG00118,HG00119", format="DP", vartype="snps",
    pass = TRUE, info = FALSE)
boxplot(vcf$DP, names=vcf$samples, ylab="Read Depth (DP)")
svfile <-  "https://ftp.1000genomes.ebi.ac.uk/vol1/ftp/data_
    collections/1000G_2504_high_coverage/working/20210124.SV_
    Illumina_Integration/1KGP_3202.gatksv_svtools_novelins.
    freeze_V3.wAF.vcf.gz"
sv <- vcfsummary(svfile, svtype = TRUE)
boxplot(sv[c("DEL","DUP", "CNV", "INS","INV","CPX","CTX")],
         main = "SVs per genome stratified by SV types")
vcffiles <- paste0("https://ftp.1000genomes.ebi.ac.uk/vol1/
    ftp/data_collections/1000G_2504_high_coverage/working/
    20201028_3202_raw_GT_with_annot/20201028_CCDG_14151_B01_
    GRM_WGS_2020-08-05_chr", 1:22, ".recalibrated_variants.
    vcf.gz")
all <- mclapply(vcffiles,
vcfsummary, pass = TRUE, mc.cores=22)
ped <- read.table("https://ftp.1000genomes.ebi.ac.uk/vol1/
    ftp/data_collections/1000G_2504_high_coverage/20130606_
    g1k_3202_samples_ped_population.txt", h=T)
ped <- ped[order(ped$Superpopulation),]
supers <- unique(ped$Superpopulation)
samples <- all[[1]]$samples
snps <- Reduce("+", lapply(all, "[[", "SNP"))
indels <- Reduce("+", lapply(all, "[[", "INDEL"))
o <- sapply(supers, function(pop) {
  id <- subset(ped, Superpopulation == pop)[,"SampleID"]
  ord <- match(id, samples)
  (snps[ord] + indels[ord]) / 1e6
})
boxplot(o, main = "SNP & INDEL with FILTER=PASS", ylab = "
    Number of variants per genome (in millions)")
```
